# Supplementary figures and images for: Next-Gen intestinal parasite detection: Leveraging metataxonomics for improved diagnosis of intestinal protists and helminths
Source: PLoS One. 2026 Jan 2;21(1):e0330312. doi: 10.1371/journal.pone.0330312 (PMC12758778; doi:10.1371/journal.pone.0330312)

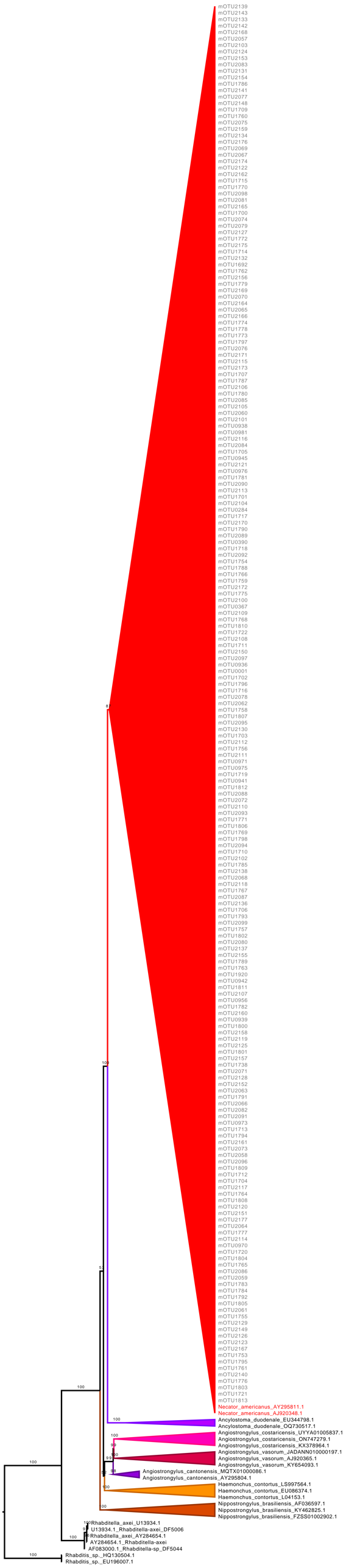

Supplement: S1 Fig — Maximum-likelihood phylogenetic tree based on the 18S rRNA gene, constructed using a curated selection of reference sequences from nematode genera related to hookworms, including representatives of Necator and Ancylostoma. The tree was inferred with 1,000 ultrafast bootstrap (UFBoot) replicates to assess branch support. Molecular OTUs (mOTUs) identified in this study are labeled with the prefix “mOTU”. All hookworm mOTUs clustered within the Necator americanus clade, indicating that this was the sole hookworm species detected in the sampled population. Ultrafast bootstrap support values are shown at the corresponding nodes. Rhabditis sp. was used as the outgroup. (PDF) [file pone.0330312.s001.pdf]

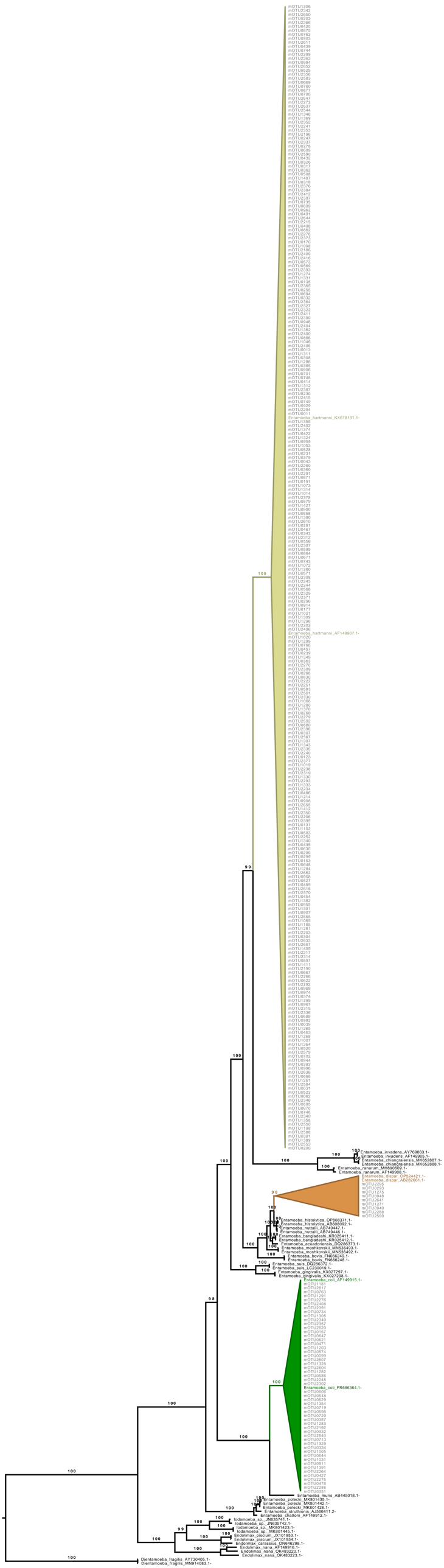

Supplement: S2 Fig — Maximum-likelihood phylogenetic tree based on the 18S rRNA gene, constructed using a curated selection of reference sequences from the genus Entamoeba. The tree was inferred with 1,000 ultrafast bootstrap (UFBoot) replicates to assess branch support. Molecular OTUs (mOTUs) identified in this study are labeled with the prefix “mOTU”. All mOTUs clustered within known Entamoeba species clades, supporting their taxonomic assignments. Ultrafast bootstrap support values are indicated at the corresponding nodes. Representative sequences of Dientamoeba fragilis were used as the outgroup. (PDF) [file pone.0330312.s002.pdf]

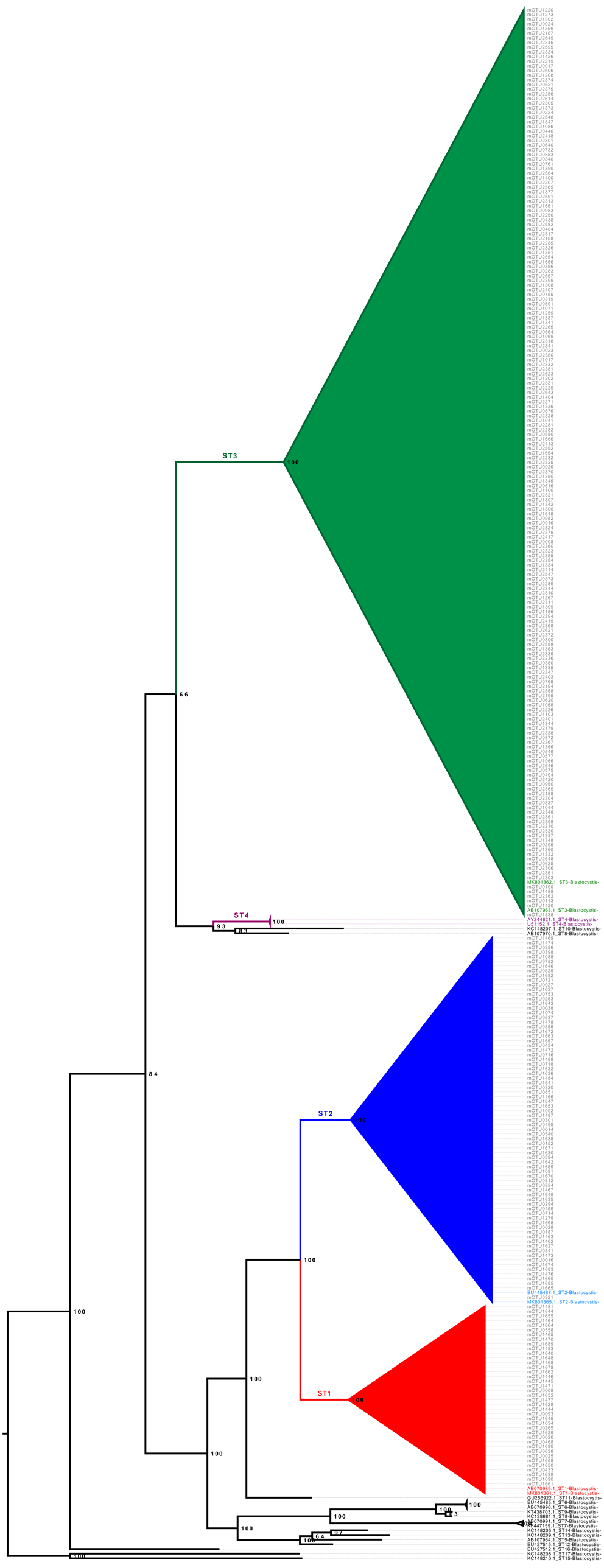

Supplement: S3 Fig — Maximum-likelihood phylogenetic tree based on the 18S rRNA gene, constructed using a curated selection of Blastocystis reference sequences representing subtypes (STs) 1–17, including all subtypes commonly associated with human populations. Molecular OTUs (mOTUs) identified in this study are labeled with the prefix “mOTU”. The tree was inferred with 1,000 ultrafast bootstrap (UFBoot) replicates to assess branch support, and UFBoot values are shown at the corresponding nodes. All mOTUs clustered within well-supported clades corresponding to known Blastocystis subtypes, enabling confident subtype assignment. A combined clade of Blastocystis ST15 and ST17 was used as the outgroup. (PDF) [file pone.0330312.s003.pdf]
