# Supplementary material for: Next-Gen intestinal parasite detection: Leveraging metataxonomics for improved diagnosis of intestinal protists and helminths
Source: PLoS One. 2026 Jan 2;21(1):e0330312. doi: 10.1371/journal.pone.0330312 (PMC12758778; doi:10.1371/journal.pone.0330312)
Supplement: Attachment — Submitted filename: ReviwersResponse_R1_FINAL.docx [file pone.0330312.s008.docx]

PONE-D-25-39373

**Next-Gen Intestinal Parasite Detection: Leveraging Metataxonomics for Improved Diagnosis of Intestinal Protists and Helminths**

**Rebuttal Letter**

R/: We are very grateful to the editor and reviewers for investing their time in carefully reviewing our manuscript and for their thoughtful comments, critiques, and recommendations. We have made every possible effort to improve the manuscript by addressing all the feedback, and we are confident that this revised version represents a significant improvement.

**1. Please ensure that your manuscript meets PLOS ONE's style requirements, including those for file naming. The PLOS ONE style templates can be found at**

[**https://journals.plos.org/plosone/s/file?id=wjVg/PLOSOne_formatting_sample_main_body.pdf**](https://journals.plos.org/plosone/s/file?id=wjVg/PLOSOne_formatting_sample_main_body.pdf) **and**

[**https://journals.plos.org/plosone/s/file?id=ba62/PLOSOne_formatting_sample_title_authors_affiliations.pdf**](https://track.editorialmanager.com/CL0/https:%2F%2Fjournals.plos.org%2Fplosone%2Fs%2Ffile%3Fid=ba62%2FPLOSOne_formatting_sample_title_authors_affiliations.pdf/1/010f01997c850ff8-89c31f94-8bba-48a2-829a-b75a66e69046-000000/819_d-Dl-Ws94hAtfr9Cj0_5Xd2eiO0GTtSl10Cz-nQ=230)**.**

**R:/** We have adjusted the manuscript format according to the journal’s recommendations and renamed the figures and supplementary materials as suggested.

**2. Thank you for stating the following financial disclosure:**

**“This study was funded by Escuela de Microbiología-CODI, Universidad de Antioquia, under grant code 2023-64370.”**

**Please state what role the funders took in the study.  If the funders had no role, please state: "The funders had no role in study design, data collection and analysis, decision to publish, or preparation of the manuscript."**

**If this statement is not correct you must amend it as needed.**

**Please include this amended Role of Funder statement in your cover letter; we will change the online submission form on your behalf.**

**R:/** Thank you for the comment. Indeed, the funders had no role in the study, and we have added this clarification to the Acknowledgments section:

“This study was funded by Escuela de Microbiología-CODI, Universidad de Antioquia, under grant code 2023-64370. The funders had no role in study design, data collection and analysis, decision to publish, or preparation of the manuscript.”

**3. Please note that your Data Availability Statement is currently missing [the repository name and/or the DOI/accession number of each dataset OR a direct link to access each database]. If your manuscript is accepted for publication, you will be asked to provide these details on a very short timeline. We therefore suggest that you provide this information now, though we will not hold up the peer review process if you are unable.**

**R:/** The data were made publicly available on October 16th, and the BioProject containing the raw data can be accessed through the following link:

https://www.ncbi.nlm.nih.gov/bioproject/PRJNA1293464

This information was also added to the manuscript.

**4. Thank you for stating the following in the Acknowledgments Section of your manuscript:**

**“This study was funded by Escuela de Microbiología-CODI, Universidad de Antioquia, under grant code 2023-64370.”**

**We note that you have provided additional information within the Acknowledgements Section that is not currently declared in your Funding Statement. Please note that funding information should not appear in the Acknowledgments section or other areas of your manuscript. We will only publish funding information present in the Funding Statement section of the online submission form.**

**Please remove any funding-related text from the manuscript and let us know how you would like to update your Funding Statement. Currently, your Funding Statement reads as follows:**

**“This study was funded by Escuela de Microbiología-CODI, Universidad de Antioquia, under grant code 2023-64370”**

**Please include your amended statements within your cover letter; we will change the online submission form on your behalf.**

**R:/** Thank you for the clarification. The funding information was removed from the Acknowledgements section and is not mentioned elsewhere in the manuscript. The funding information provided in the online submission form should be as follows:

This study was funded by Escuela de Microbiología-CODI, Universidad de Antioquia, under grant code 2023-64370. The funders had no role in study design, data collection and analysis, decision to publish, or preparation of the manuscript.

**5. We note that there is identifying data in the Supporting Information file <Supp_TABLE_1_NGS_Mic.xlsx>. Due to the inclusion of these potentially identifying data, we have removed this file from your file inventory. Prior to sharing human research participant data, authors should consult with an ethics committee to ensure data are shared in accordance with participant consent and all applicable local laws.**

**Data sharing should never compromise participant privacy. It is therefore not appropriate to publicly share personally identifiable data on human research participants. The following are examples of data that should not be shared:**

**-Name, initials, physical address**

**-Ages more specific than whole numbers**

**-Internet protocol (IP) address**

**-Specific dates (birth dates, death dates, examination dates, etc.)**

**-Contact information such as phone number or email address**

**-Location data**

**-ID numbers that seem specific (long numbers, include initials, titled “Hospital ID”) rather than random (small numbers in numerical order)**

**Data that are not directly identifying may also be inappropriate to share, as in combination they can become identifying. For example, data collected from a small group of participants, vulnerable populations, or private groups should not be shared if they involve indirect identifiers (such as sex, ethnicity, location, etc.) that may risk the identification of study participants.**

**Additional guidance on preparing raw data for publication can be found in our Data Policy (**[**https://journals.plos.org/plosone/s/data-availability#loc-human-research-participant-data-and-other-sensitive-data**](https://journals.plos.org/plosone/s/data-availability#loc-human-research-participant-data-and-other-sensitive-data)**) and in the following article:** [**http://www.bmj.com/content/340/bmj.c181.long**](http://www.bmj.com/content/340/bmj.c181.long)**.**

**Please remove or anonymize all personal information (<specific identifying information in file to be removed>), ensure that the data shared are in accordance with participant consent, and re-upload a fully anonymized data set. Please note that spreadsheet columns with personal information must be removed and not hidden as all hidden columns will appear in the published file.**

**R:/** Thanks for the clarification. The table S1 was modified to remove any information related to age, sex, or any other details that could compromise participant privacy.

**6. If the reviewer comments include a recommendation to cite specific previously published works, please review and evaluate these publications to determine whether they are relevant and should be cited. There is no requirement to cite these works unless the editor has indicated otherwise.**

**R:/** Thanks for the observation. We took it into consideration while preparing the revised version of the manuscript.

**Additional Editor Comments:**

**The claim that metataxonomics outperforms microscopy is presented too strongly. The results show that sequencing was superior for detecting Strongyloides and Blastocystis, yet microscopy was more effective for Trichuris and Ascaris. This contrast demonstrates that the performance of each method is parasite dependent. The interpretation should therefore highlight complementarity rather than positioning one method as superior to the other.**

**The treatment of discrepancies between methods is not sufficiently critical. For example, the lack of Giardia detections in sequencing is attributed to true absence in the population, yet microscopy found positive cases. Technical limitations such as primer mismatch or inefficient DNA extraction provide more plausible explanations. Similar issues appear for Iodamoeba and Cyclospora. The manuscript would benefit from a stronger discussion of methodological causes for these mismatches, ideally supported by targeted molecular validation.**

**R:/** We fully agree with the comment and have revised the manuscript to present the two methods as complementary. Our main idea is that this technology represents a new tool within the parasitologist’s technological toolbox. Additionally, we have included the following paragraph in the manuscript to further elaborate on the possible explanations for the incongruent results observed between the NGS and microscopy analyses:

The most discordant results were observed for other protozoa, namely *Chilomastix*, *Cyclospora*, *Cystoisospora*, and *Giardia*. For these protozoan taxa, there was no concordance between microscopy and NGS metataxonomic; each was detected by only one method, with no mutual confirmation. This highlights the inherent challenge of capturing the full diversity of parasites using a single methodological approach, given the varying biological and molecular characteristics of these organisms. In 18S rRNA metataxonomics, several technical factors may contribute to false-negative results. First, DNA extraction protocols may not fully disrupt the resistant cyst or oocyst walls of certain protozoa, limiting the recovery of amplifiable DNA (Naushad et al., 2025). Second, sequence polymorphisms in the target regions can affect the binding efficiency of universal primers, introducing amplification bias (Bradley et al., 2016). Third, the low abundance of parasite DNA relative to host and microbiome DNA can reduce amplification efficiency (Frau et al., 2019). Additionally, variability in ribosomal gene copy number among taxa and preferential amplification of shorter fragments, particularly when targeting V3-V4 regions, can further affect detection sensitivity (Chihi et al., 2022; Stensvold, 2024). Taken together, these factors indicate that absence of detection by NGS should be interpreted cautiously, as it may reflect technical limitations rather than true absence of the organism.

In the case of *Chilomastix*, bioinformatic analysis revealed an issue similar to that observed with *Iodamoeba*: the reverse primer target region shows poor sequence conservation in this genus, likely impairing efficient primer annealing and thus detection by PCR. For *Cyclospora*, although we cannot entirely rule out primer incompatibility, the same primer set has successfully amplified other apicomplexan parasites (e.g., *Cystoisospora*, *Cryptosporidium*) in past analyses. Therefore, no definitive conclusion can be made regarding *Cyclospora* detection.

Interestingly, *Giardia* was not detected by NGS in any of the samples analyzed in this study, even though we have previously reported its successful detection using the same primers and protocols in human stool samples and wastewater from Colombia (Rozo-Montoya et al., 2023). This observation is consistent with other metataxonomic studies that have reported limited or no detection of *Giardia*, highlighting persistent technical challenges (Ramayo-Caldas et al., 2020; Stensvold et al., 2021; Wylezich et al., 2019, 2020). Several authors have suggested that primer mismatches, low abundance, and especially inefficient DNA extraction from the parasite’s resistant cyst wall may underline these failures. Standard fecal DNA extraction protocols may be suboptimal for *Giardia* which often require more intensive lysis methods— such as mechanical disruption or repeated freeze-thaw cycles— to ensure adequate DNA release (Babaei et al., 2011).

**The sample size of 65 is small and combines specimens from two time periods almost seven years apart. Pooling across such a wide temporal gap introduces the possibility of confounding through epidemiological changes or laboratory practice differences. The authors should address this explicitly and discuss how these potential biases were controlled or accounted for in the study design.**

**R:/** We thank the reviewer for raising these relevant points. While the cohort of 65 samples may appear limited, it was sufficient to capture the diversity of intestinal parasites circulating in the studied populations and to demonstrate the applicability and performance of the metataxonomic–phylogenetic framework. Our primary objective was methodological—to evaluate the capacity of NGS-based metataxonomics to detect and resolve multiple intestinal parasites within real field samples—rather than to perform a large-scale epidemiological survey. Moreover, we fully acknowledge the potential implications of combining samples collected at different time periods. Our primary objective was to perform a methodological comparison between NGS and microscopy for parasite detection in an endemic setting. The relative stability of parasitic infections in such regions provides a suitable context in which pooling these samples enables a robust evaluation of the technique’s capacity to reveal diversity that might be overlooked by microscopy alone. Moreover, it is important to note that all stool samples were processed within a short time after collection—within a few days—and DNA extraction was performed using the same commercial kit by the same highly trained Ph.D.-level researcher experienced in molecular biology techniques. All purified DNA samples were stored at −80 °C to ensure optimal preservation and minimize degradation. These steps were taken to control potential biases as much as possible. In response to this comment, we will add a paragraph to the discussion explicitly addressing how long-term sample storage could affect DNA quality and NGS performance, thereby strengthening the interpretation of our findings:

“Another potential limitation of this study is the long-term storage of DNA samples prior to NGS analysis. While prolonged preservation at -80^o^C generally maintains DNA integrity, factors such as storage duration and freeze-thaw cycles can influence quality. Evidence from microbiota research indicates that fecal DNA can remain viable for extended periods, even up to 14 years, under stable frozen conditions (Kia et al., 2016; Kim et al., 2023; Nel Van Zyl et al., 2020). However, parasite specific data is more limited. Although studies suggest that parasite DNA, such as from *Giardia*, remains detectable after long-term storage (Kuk et al., 2012), the effect on the broader parasitome is less clear. In our study, all samples were maintained under a strict and uniform storage protocol at -80^o^C from collection until DNA extraction. This consistent handling ensures that any potential DNA degradation would be systematic across the entire sample set. Consequently, such effects would likely reduce overall detection sensitivity uniformly, without compromising our core finding that NGS identifies greater parasite diversity than microscopy in these archival samples.”

**Figures showing detection frequencies are presented as simple bar charts without any measure of uncertainty. Although presence and absence data are used, statistical analysis or variability indicators would allow readers to assess the strength of the comparisons. In the phylogenetic analyses, bootstrap values are presented as definitive, yet even high values need careful interpretation. A more cautious discussion of these analyses is necessary.**

**R:/** We sincerely thank the reviewer for this thoughtful and constructive observation. We fully agree that including measures of uncertainty and adopting a more cautious interpretation of the phylogenetic analyses will strengthen the manuscript.

It is important to note that our main goal in this work is to demonstrate how classical parasitological methods can be complemented by NGS-based metataxonomics and to highlight the potential limitations inherent to each approach. We believe that the results presented here will open new avenues for research in diagnostic methodologies, and that the conclusions drawn from this study will help guide the design of more quantitative and statistically robust analyses in future investigations.

Regarding the phylogenetic analyses, we have carefully revised the text to moderate our interpretation of bootstrap support values, emphasizing that—even when high—these values represent statistical confidence within the dataset and should not be regarded as absolute indicators of phylogenetic certainty. As an additional line of support, the selection of molecular operational taxonomic units (mOTUs) corresponding to each genus was validated through BLASTN comparisons against reference sequences. These comparisons consistently showed nearly full-length alignments of the mOTUs to their respective references, with nucleotide identity values exceeding 99%, reinforcing the accuracy of our taxonomic assignments. Nonetheless, we acknowledge that ribosomal markers offer limited phylogenetic resolution. Future studies should therefore adopt phylogenomic approaches to achieve greater confidence and resolution, which will enable a more accurate depiction of the true genetic diversity within intestinal parasites. Despite these constraints, ribosomal genes remain the current reference framework for metataxonomic analyses and the principal taxonomic standard in parasitology.

We have modified several paragraphs in the discussion highlighting this point.

**The methods section relies on a single primer pair targeting the 18S V4 region, which has known limitations. The exclusion of cestodes, trematodes, and some amoebae is acknowledged briefly, but the manuscript still describes the method as broad spectrum. A more nuanced description of primer biases and their impact on detection outcomes would improve the study’s credibility.**

**R:/** We fully agree that the 18S rRNA V4 primer pair employed in our study has known amplification biases that can limit the detection of certain parasite taxa, particularly cestodes, trematodes, and some amoebae. In the revised manuscript, we have clarified this limitation more explicitly in both the *Discussion* section.

**The raw sequencing data are stated to be deposited in the NCBI SRA under BioProject accession PRJNA1293464, with a reviewer link provided. This is an important positive step, and the final version should clearly include accession numbers to ensure compliance with data availability policies and to allow independent verification of results.**

**R:/** The data were made publicly available on October 16th, and the BioProject containing the raw data can be accessed through the following link:

https://www.ncbi.nlm.nih.gov/bioproject/PRJNA1293464

This information was also added to the manuscript.

**The discussion emphasizes advantages of sequencing approaches, yet does not sufficiently acknowledge the limitations of this marker or the need for complementary methods. For instance, species level identification of Ascaris is not possible with 18S rDNA, yet the text does not consistently highlight this limitation. Balancing strengths with weaknesses would provide a more rigorous and realistic assessment of the approach.**

**R:/** We thank the reviewer for this valuable comment and fully agree that the Discussion should more explicitly acknowledge the limitations of using the 18S rDNA marker and the need for complementary methods. In the revised version, we have clarified that while 18S rDNA-based metataxonomics provides a powerful framework for a broader detection and preliminary taxonomic assignment, it lacks sufficient resolution for species-level discrimination in certain taxa, and that confirmatory analyses require multilocus (MLSA) or full phylogenomic approaches.

We have added statements in the Discussion emphasizing that future studies should incorporate phylogenomic or multilocus strategies to improve confidence in species-level identification and to capture deeper evolutionary relationships. Nonetheless, we consider that despite these limitations, ribosomal markers will remain, for the foreseeable future, an accepted reference framework for metataxonomic analyses and will continue to foster progress in parasitology.

Modified paragraph in discussion section:

“One potential advantage of the metataxonomic approach lies in its capacity, when integrated with phylogenetic analyses, to provide putative species-level assignments for several intestinal parasites. However, such classifications should be interpreted with caution, as they are limited by the phylogenetic resolution of the ribosomal marker used and by the completeness of current taxonomic reference databases. As NGS technologies continue to advance and genomic databases for parasites become more comprehensive, the inclusion of multiple molecular markers—or even full phylogenomic approaches—will enable more confident species-level identifications within a robust and resolved taxonomic framework.”

**Ethical approval is reported and the use of archived anonymized samples is clearly justified. Consent and confidentiality procedures are explained adequately. However, the quality control procedures for microscopy are not described in detail. Including information on slide reading standards and technician validation would help rule out operator variability as a source of discrepancy.**

**R:/** We appreciate the reviewer’s suggestion to improve the clarity of our methods. We agree that a detailed description of the microscopy quality control is essential to rule out operator variability as a significant source of discrepancy. In response, we have thoroughly revised the Sample description and microscopic diagnosis of the Methods section to provide a comprehensive account of our procedures:

**“Sample description and microscopic diagnosis**

A total of 65 fecal samples were selected in the present study based on confirmed microscopic diagnoses of the soil transmitted helminths and protists. Fecal samples were obtained from individuals in selected regions of Colombia as part of previous research projects. At the time of collection, stool samples were processed using direct saline and iodine smears, following a concentration step using the MiniParasep® SF fecal parasite concentrator (Apacor Ltd, England). The microscopic examination was conducted by an experienced staff of professionals with specific expertise in intestinal parasite diagnosis, following standardized internal protocols aligned with clinical laboratory guidelines (Garcia et al., 2018). To ensure maximum sensitivity, the entire surface area of each prepared slide (both direct and concentrated) was systematically examined under 100x and 400x magnification. The competence of the technical staff was rigorously maintained through a continuous quality assurance system that included periodic validation of diagnostic skills using reference slide collections. Additionally, a senior parasitologist was available for case consultation and verification of uncertain morphological identifications. This approach ensured standardized morphological identification and consistent performance across all operators. Samples were stored at -80ºC until DNA extraction. Prior to molecular analysis, all samples were anonymized and coded to ensure the confidentiality of participants. A complete description of the data collected, and the parasitological results is presented in S1 Table.”

**The text is clearly structured and generally readable, yet it often repeats similar arguments and adopts a promotional tone. The writing would benefit from greater conciseness, less redundancy, and a more neutral presentation of the study’s impact. This would align the manuscript more closely with the standards of scientific reporting.[Note: HTML markup is below. Please do not edit.]Reviewers' comments:**

**R:/** We sincerely thank the reviewer for this valuable feedback. We have carefully revised the manuscript to improve clarity, reduce redundancy, and adopt a more neutral and objective tone throughout. Repetitive statements emphasizing methodological advantages or interpretive claims have been condensed or removed to ensure a more concise and balanced presentation that aligns with scientific reporting standards. We believe these edits have substantially improved the manuscript’s readability and overall scientific tone.

Reviewer's Responses to Questions

**Comments to the Author**

**1. Is the manuscript technically sound, and do the data support the conclusions? The manuscript must describe a technically sound piece of scientific research with data that supports the conclusions. Experiments must have been conducted rigorously, with appropriate controls, replication, and sample sizes. The conclusions must be drawn appropriately based on the data presented.**

**Reviewer #1:Yes**

**Reviewer #2:Partly**

**2. Has the statistical analysis been performed appropriately and rigorously?**

**Reviewer #1:N/A**

**Reviewer #2:Yes**

**3. Have the authors made all data underlying the findings in their manuscript fully available?**

**The** [**PLOS Data policy**](http://track.editorialmanager.com/CL0/http:%2F%2Fwww.plosone.org%2Fstatic%2Fpolicies.action%23sharing/1/010f01997c850ff8-89c31f94-8bba-48a2-829a-b75a66e69046-000000/HJ2VWEDjJ61kuqkPWTAqT-My2AY2H-65_GIxQtJi6XA=230) **requires authors to make all data underlying the findings described in their manuscript fully available without restriction, with rare exception (please refer to the Data Availability Statement in the manuscript PDF file). The data should be provided as part of the manuscript or its supporting information, or deposited to a public repository. For example, in addition to summary statistics, the data points behind means, medians and variance measures should be available. If there are restrictions on publicly sharing data—e.g. participant privacy or use of data from a third party—those must be specified.**

**Reviewer #1:Yes**

**Reviewer #2:Yes**

**4. Is the manuscript presented in an intelligible fashion and written in standard English?
PLOS ONE does not copyedit accepted manuscripts, so the language in submitted articles must be clear, correct, and unambiguous. Any typographical or grammatical errors should be corrected at revision, so please note any specific errors here.**

**Reviewer #1:Yes**

**Reviewer #2:Yes**

**5. Review Comments to the Author**

**Please use the space provided to explain your answers to the questions above. You may also include additional comments for the author, including concerns about dual publication, research ethics, or publication ethics. (Please upload your review as an attachment if it exceeds 20,000 characters)**

**R:/** We thank the reviewer for the constructive recommendations that allowed us to significantly improve the manuscript. The new version has been carefully revised to address all comments and concerns raised by both the reviewers and the editor. We hope you will find this revised version satisfactory and adequately improved.

**Reviewer #1:This is an interesting manuscript which describes the use of NGS for identifying parasites in clinical samples, focusing on Blastocystis and Entamoeba spp. The manuscript is well written and follows a logical structure, making it easy for readers to follow the flow of the study. The technical terminology used throughout is appropriate and no major grammatical errors were identified.**

**I felt that the introduction could be expanded to include more recent studies on NGS in parasitology but otherwise provided a good overview of the wider literature within the context of parasitology and molecular diagnostics.**

**R:/** We thank the reviewer for this positive feedback and valuable suggestion. In direct response to the comment, we have expanded the Introduction to incorporate a more detailed overview of recent studies applying NGS in parasitology:

“Microscopy-based methods are the common standard for intestinal parasite detection due to their low cost and simplicity (Momčilović et al., 2019). However, they exhibit low sensitivity and specificity which is highly dependent on the parasite load and technician expertise (Miswan et al., 2022). They also lack the resolution needed to accurately differentiate morphologically similar species, such as *Entamoeba histolytica* from non-pathogenic *Entamoeba* spp. (Carrero et al., 2020), *Ascaris lumbricoides* from closely related zoonotic species like *Ascaris suum* (Betson et al., 2014), or among human-infecting hookworms such as *Necator americanus* and *Ancylostoma duodenale* (Wang et al., 2012). Consequently, there is a growing need for studies using advanced diagnostic tools to accurately identify parasite species infecting human and animal populations in specific areas (Hassan et al., 2021; Khurana et al., 2021a; Rodpai et al., 2018).

To overcome these limitations, PCR-based methods have been widely adopted, offering superior sensitivity and specificity for detecting specific parasite species or genera (Khurana et al., 2021b; Verweij & Stensvold, 2014). A significant advance has been the development of multiplex PCRs for the simultaneous detection of several parasites in a single reaction (Autier et al., 2018; Garg et al., 2024; Khurana et al., 2021b; Lamien-Meda et al., 2020; Llewellyn et al., 2016). However, these molecular approaches present their own challenges. Current multiplex PCR assays for intestinal parasites exhibit significant limitations in their target selection, creating diagnostics gaps that may compromise comprehensive parasitological assessment. These assays are specifically designed to detect established pathogenic species, most of them protozoa, systematically excluding helminths and non-pathogenic organisms.

Next-generation sequencing (NGS) strategies emerge as a powerful alternative, enhancing taxonomic resolution through untargeted detection and comprehensive characterization of parasite diversity. These approaches overcome the limitations of specific PCR methods by enabling the simultaneous identification of multiple taxonomic groups, either via direct DNA sequencing (metagenomic) or PCR-based amplification of specific genetic markers (metataxonomic) (Chourasia et al., 2025; Kounosu et al., 2019; Wylezich et al., 2020). Metataxonomic approaches have become invaluable in microbial ecology due to their cost-effectiveness and capacity to identify a broad range of organisms within a single sample. These studies typically target ribosomal RNA gene regions, such as 16S for bacteria, 18S for eukaryotes, and the internal transcribed spacer (ITS) region (Breitwieser et al., 2019). While widely applied in bacterial research, metataxonomic analysis in parasitology remains limited. Applications in protists have shown promising results (Garcia-Montoya et al., 2023; Hino et al., 2016; Moreno et al., 2018; Rusiñol et al., 2020; Stensvold et al., 2020; Zahedi et al., 2019), yet its implementation for human associated helminth detection is still scarce (Lappan et al., 2019). In protist research, it has revealed greater diversity than traditional methods, enabling the identification of mixed infections and novel genetic variants of parasites like *Blastocystis* (Garcia-Montoya et al., 2023b; Higuera et al., 2021; Maloney et al., 2019; Maloney, Molokin, da Cunha, et al., 2020), as well as the detection of various intestinal protists (*Entamoeba*, *Iodamoeba*, *Balantioides*, *Tetrathrichomonas*) and taxa typically excluded from routine diagnostics, such as *Dientamoeba* (Wylezich et al., 2019, 2020). In helminths, NGS has been validated for taxonomic classification, demonstrating efficacy in diverse contexts from soil nematode biodiversity to the identification of key soil-transmitted helminths (STH) including *Ascaris*, *Trichuris*, and *Strongyloides* in modern and archaeological samples (Chessa et al., 2020; Kenmotsu et al., 2020, 2021; Tanaka et al., 2014), as well as trematodes such as *Schistosoma* spp. and cestodes like *Taenia* spp. and *Echinococcus* spp. (Basirpour et al., 2025). Together, these findings demonstrate that sequencing-based approaches not only mitigate the limitations of traditional methods but also opens new pathways for the discovery of unexpected pathogens.

In response to the recognized diagnostic gaps in parasitology, this study explores the application of a metataxonomic approach coupled with phylogenetic methods for the simultaneous detection and, when possible, species-level confirmation of multiple parasite taxa in human fecal samples from Colombia. By evaluating a scalable, high-throughput strategy, the study aims to contribute to ongoing surveillance efforts and support more accurate monitoring in endemic settings.”

**The discussion was generally well structured but could benefit from the following minor revisions:**

**1) There is no mention of sequencing depth and coverage. Was this sufficient to detect low-abundance parasites? Insufficient sequencing depth could lead to under-detection of rare species or mixed infections. The authors should discuss how sequencing variability might influence the results.**

**R:/** We appreciate the reviewer’s insightful comment. We agree that sequencing depth and coverage are key factors influencing the detection of low-abundance taxa and mixed infections in amplicon-based metataxonomic studies. In our dataset, the number of raw paired-end reads per sample ranged from **64,254 to 214,280**, and after Q30 quality filtering, from **31,891 to 120,273** reads. Following Mothur-based quality control, the number of high-quality sequences retained per sample ranged from **5,884 to 109,502**, with **Good’s coverage values between 0.996019 and 0.99997**, indicating near-complete sampling of the dominant taxa present.

We acknowledge, however, that despite the high sequencing coverage achieved, the detection of very low-abundance taxa may have been limited by amplification biases and stochastic effects inherent to PCR-based approaches. Nonetheless, the consistently high coverage values observed in our dataset suggest that the sequencing depth was likely sufficient to provide an acceptable level of sensitivity for detecting the main parasitic taxa, which was one of the primary goals of this study.

In the revised manuscript, we have now included a detailed description of the sequencing coverage results in the Methods section:

“Sequencing depth and quality were evaluated across all samples to ensure adequate coverage for metataxonomic analyses. The number of raw read pairs per sample ranged from **64,254 to 214,280**, with post-filtering Q30-cleaned read pairs ranging from **31,891 to 120,273**. Read cleaning was performed with cutadapt v2.10. After applying the Mothur quality-control pipeline, the number of high-quality sequences retained per sample ranged from **5,884 to 109,502**. The estimated Good’s coverage values varied between **0.996019 and 0.99997**, indicating that the sequencing effort was sufficient to capture nearly the entire diversity of dominant taxa present in each sample.”

**2) The potential risk of contamination during sample processing or sequencing is not discussion. This is a common issue in NGS studies, potentially leading to false positives or biased results.**

**R:/** We thank the reviewer for this valuable observation and fully agree that contamination and cross-sample carryover represent important challenges in NGS-based studies, potentially leading to false positives or spurious detections. In our study, several measures were implemented to minimize this risk. DNA extractions and PCR setups were conducted in physically separated pre-PCR laboratories using dedicated reagents and filtered tips to prevent cross-contamination. Additionally, within the same batch of samples, we included food samples of unrelated origin as negative controls, which consistently tested negative for human parasite sequences. Moreover, as observed by the marked differences in parasite composition and diversity across individual fecal samples suggest that cross-contamination did not influence our results.

3) **How can this be translated to clinical practice? Are the identified parasites clinically significant? What is the practicality of implementation in routine clinical/ field settings?**

**R:/** Thank you for your insightful comment. In terms of clinical relevance, several of the parasites identified in our study —such as *Strongyloides stercoralis*, *Ascaris lumbricoides*, *Trichuris trichiura*, several *Blastocystis* subtypes, and *Necator americanus* — are well-established pathogens or organisms of uncertain but emerging clinical significance. *Strongyloides stercoralis*, in particular, is of high medical importance due to its potential for life-threatening hyperinfections, especially in immunocompromised patients. The ability of metataxonomics to detect such infections more sensitively than microscopy underscores its potential for improving patient outcomes through earlier or more accurate diagnosis.

Regarding practical implementation, we acknowledge that widespread adoption of NGS-based diagnostics in routine clinical and field settings still faces logistical challenges, including cost, infrastructure, and turnaround time. However, as sequencing technologies continue to become more affordable and portable (e.g., through small scale platforms), and as streamlined bioinformatics pipelines become more accessible, the use of targeted NGS in reference laboratories and surveillance programs is increasingly feasible. Importantly, the ability to detect multiple parasites (and subtypes) from a single stool sample using universal primers reduces the need for multiple diagnostic tests, making it a promising tool for high-throughput settings.

Finally, we recognized current technical limitations related to DNA extraction challenges, as observed with helminths like *Trichuris trichiura* and protozoan like *Giardia*. These issues highlight the importance of optimizing sample preparation and primer design.

4) **Discuss any recent studies on NGS applications in parasitology (if any). Include a more critical comparison of NGS with other emerging diagnostic technologies.**

**R:/** Thank you for this valuable suggestion. In response, we will include a new paragraph in the revised manuscript summarizing recent studies that apply next-generation sequencing (NGS) in parasitology, with particular emphasis on its use for detecting both protozoan and helminth parasites.

“Next-generation sequencing (NGS) has not replaced conventional diagnostic tools but has experienced growing adoption as a complementary methodology in parasitology. Its applications have expanded significantly beyond detection to include understanding genetic interrelationships among parasites, characterizing genetic diversity and intra-isolate variation, and determining the relative abundance of specific parasite species within complex clinical samples (Stensvold, 2024). The available research demonstrates several key comparative advantages of NGS over traditional diagnostic tools for parasite detection, though comprehensive head-to-head studies remain limited. When comparing next-generation sequencing (NGS) to PCR in the diagnosis of parasitic infections, NGS offers broader detection capabilities, particularly for mixed infections and subtype resolution, while PCR tends to provide higher sensitivity for single-target detection. For example, in studies on *Blastocystis* spp., qPCR was more sensitive for detecting the presence of the parasite in low-load samples, but NGS was superior in identifying multiple subtypes and mixed infections, which were undetected by PCR and Sanger sequencing (Šloufová et al., 2022). Stensvold et al. directly compared metabarcoding (NGS) and real-time PCR (qPCR) for detecting zoonotic protists in pigs, revealing complementary methodological strengths (Stensvold et al., 2021). While both methods were equally effective for detecting *Balantioides coli*, metabarcoding proved superior for broad-spectrum screening, simultaneously identifying additional parasites like *Giardia intestinalis* and *Enterocytozoon bieneusi*. In contrast, qPCR remained the optimal tool for sensitive, targeted quantification of specific pathogens. In relation to intestinal amoebae, several studies have demonstrated that next-generation sequencing (NGS) offers markedly improved sensitivity and resolution over conventional methods such as microscopy and PCR. NGS-based approaches, including amplicon sequencing and metagenomics, consistently detect higher species richness, mixed infections, and intra-host diversity that are frequently overlooked by traditional diagnostics (Guilane et al., 2024; Stensvold, Ascuña‐Durand, et al., 2023; Stensvold, Berg, et al., 2023). For instance, short-amplicon NGS revealed substantial genetic diversity within *Iodamoeba bütschlii*, including host-specific lineages and novel variants undetectable by standard PCR (Guilane et al., 2024). Similarly, NGS analyses of *Entamoeba coli*, *E. hartmanni*, and *Endolimax nana* have uncovered extensive intrageneric diversity and previously unrecognized ribosomal lineages (Hocke et al., 2022; Stensvold, Ascuña‐Durand, et al., 2023). In livestock and wildlife hosts, NGS has identified mixed-subtype infections and novel genotypes of *Entamoeba* and *Blastocystis*, expanding the known host range and genetic landscape of these protists (Stensvold, Berg, et al., 2023; Wylezich et al., 2019).

NGS has proven particularly valuable for protozoan pathogens such as *Giardia intestinalis* and *Cryptosporidium* spp., where mixed infections are common but often undetected by conventional sequencing methods. For *Giardia intestinalis*, several studies have demonstrated that next-generation amplicon sequencing (NGS) provides significant methodological advantages over conventional Sanger sequencing for molecular characterization and epidemiological investigations (Hublin et al., 2022; Maloney, Molokin, & Santin, 2020; Ogbuigwe et al., 2022). NGS consistently shows higher sensitivity and improved capacity to detect mixed assemblage infections, which are frequently missed or yield ambiguous results with Sanger sequencing due to its inability to resolve heterogeneous sequences. By targeting loci such as the beta-giardin and tpi genes, deep amplicon sequencing has revealed the presence and relative abundance of multiple assemblages (e.g., A, B, AII, BIV) within individual hosts and outbreak samples (Hublin et al., 2022; Maloney, Molokin, & Santin, 2020; Ogbuigwe et al., 2022). Notably, in outbreak investigations, NGS successfully identified mixed assemblages in 43.5% of cases where Sanger failed and further detected 12 novel sub-assemblage-level variants (Ogbuigwe et al., 2022). Additionally, its ability to quantify minor single-nucleotide variants present at frequencies as low as 5% provides unprecedented resolution into parasite population structure. These capabilities not only offer deeper genetic insights but also enhance the precision of transmission tracking and public health surveillance efforts. Furthermore, in the case of *Cryptosporidium*, NGS offers a critical advantage by revealing a complex landscape of infection that remains hidden to conventional methods. While Sanger sequencing of the gp60 gene often identifies only a dominant subtype, NGS applications consistently uncover mixed infections and substantial within-subtype genetic diversity (Bailly et al., 2022; Zahedi et al., 2017, 2019b).This high-resolution view of the pathogen population is crucial for accurate epidemiological tracing and for understanding the true dynamics of transmission.”

Reviewer #2: Intestinal parasites cause a major health burden worldwide, and traditional microscopy offers only limited sensitivity. In this manuscript, the authors applied an NGS-based metataxonomic approach, which showed higher sensitivity than microscopy for Strongyloides stercoralis but lower for Trichuris trichiura. The method also provided improved species- and subtype-level resolution. Overall, metataxonomics appears powerful for broad parasite detection, although primer design still requires refinement. I find this topic very interesting, but I have a few questions and suggestions to consider:

Major comments:

1. It is unclear whether random primers were used for library construction and sequencing, or whether DNA extraction was followed by library preparation employing only the 18S-V4Fw and 18S-V4 primers. An alternative possibility is that these primers were used specifically as supplements for sequencing. Clarification of this methodological step would be very helpful.

**R:/** We thank the reviewer for this insightful comment and the opportunity to clarify our methodology. In this study, library preparation was based exclusively on the amplification of the 18S rDNA V4 region using the primer pair 18S-V4Fw and 18S-V4Rev, as indicated in the Methods section. No random primers were employed at any stage of library construction. The resulting amplicons were then used directly for library preparation following Illumina’s standard amplicon workflow at Macrogen (Seoul, Korea), where indexing adapters were ligated during the library preparation process. Thus, the sequencing specifically targeted the 18S V4 amplicons generated with these primers, ensuring that all reads correspond to the selected marker region and that no random-priming or untargeted sequencing was performed. This information was updated in the methods section.

2. NGS is undoubtedly a powerful tool for species identification, but it also has the issue of noisy sequencing reads. The main concern here is that the ground truth of this study is not clearly defined, making it difficult to evaluate what are false positives or false negatives (e.g., lines 245–248) and how these were ruled out. In turns, it becomes challenging to assess which method truly outperforms the others.

**R:/** We thank the reviewer for raising this important concern regarding sequencing noise. We fully agree that distinguishing true biological signals from sequencing artifacts is a critical step in NGS-based analyses.

To address this, our workflow incorporated multiple layers of quality control to minimize the inclusion of noisy or artifactual reads. First, raw reads were quality-filtered using Cutadapt v2.10 to remove low-quality bases, retaining only high-confidence read pairs (Q30 or higher). Subsequently, the Mothur v1.44 pipeline was employed to merge cleaned paired-end reads, discard sequences containing ambiguous bases, excessive homopolymers (>8 bp), or lengths below 250 bp, and eliminate chimeric sequences detected with VSEARCH.

The overall Good’s coverage values (0.996–0.99997) demonstrate that the sequencing depth was sufficient to capture nearly all dominant taxa, minimizing stochastic detection bias. Importantly, the taxonomic assignments were further validated by phylogenetic analyses using reference sequences and BLASTN similarity searches (>99% identity) to confirm the reliability of detected taxa.

While we recognize that amplicon-based approaches inherently carry a risk of low-level noise and potential false positives, these rigorous filtering and validation steps substantially reduce such risks. Moreover, the observed consistency between NGS detections and microscopy findings for the dominant taxa (e.g., *Trichuris trichiura* and *Necator americanus*) supports the robustness of the results.

3. The authors suggest that the failure of the NGS-based metataxonomic approach for Trichuris trichiura detection may be due to the high resistance of the eggshell, which leads to the issue of DNA isolation and lower yield. This hypothesis could be easily tested by using a bioanalyzer or Qubit to precisely quantify the amount and quality of DNA.

**R:/** We agree with the reviewer and are planning to perform the proposed test to identify a more effective strategy for lysing helminth eggshells. *Trichuris trichiura* eggs are well known for their thick shell, which confers strong resistance to both mechanical and chemical lysis. In our study, DNA yield and purity were routinely assessed using a Nanodrop 2000c spectrophotometer to ensure sufficient template for downstream amplification; however, we acknowledge that fluorometric or electrophoretic quantification methods such as Qubit or Bioanalyzer would provide a more accurate measure of DNA integrity and concentration, particularly for evaluating extraction efficiency from such resistant structures. We fully agree that incorporating these analyses in future work would allow us to empirically test this hypothesis and better quantify the relationship between DNA yield and detection sensitivity in *T. trichiura* and other helminths with resistant eggshells.

Minor comments:

1. There are quite a number of typos, which require careful correction

**R:/** We appreciate the reviewer’s observation. The entire manuscript has been carefully revised to correct typographical and grammatical errors and to improve overall clarity and readability.

2. I also suggest that the authors improve the organization of their result figures and captions related to the phylogenetic analyses. (e.g., what the numbers represent? what the distinction is between “GE_Strongyloides_stercoralis_” and “Strongyloides_stercoralis” within the same phylogenetic tree??). In its current form, these figures are somewhat difficult to interpret.

**R:/** We thank the reviewer for this helpful suggestion. All phylogenetic figures and their corresponding captions have been carefully reviewed and reorganized to improve clarity and interpretability. Explanations regarding the meaning of numerical values (e.g., support values) are now clearly presented in the figure legends and Results section. The prefix “GE” referred to the origin of the reference sequence, specifically indicating sequences derived from *de novo* assembled genomes. For clarity, these prefixes were removed, and only scientific names are now used in the figures.
