# Supplementary material for: Next-Gen intestinal parasite detection: Leveraging metataxonomics for improved diagnosis of intestinal protists and helminths
Source: PLoS One. 2026 Jan 2;21(1):e0330312. doi: 10.1371/journal.pone.0330312 (PMC12758778; doi:10.1371/journal.pone.0330312)
Supplement: Attachment — Submitted filename: vANA_Dic_Rev_Response_R3.docx [file pone.0330312.s010.docx]

PONE-D-25-39373

Next-Gen Intestinal Parasite Detection: Leveraging Metataxonomics for Improved

Diagnosis of Intestinal Protists and Helminths

Rebuttal Letter

1- Journal Requirements:

If the reviewer comments include a recommendation to cite specific previously published works, please review and evaluate these publications to determine whether they are relevant and should be cited. There is no requirement to cite these works unless the editor has indicated otherwise.

Additional Editor Comments:

The references need to be double-checked, unnecessary Spanish words removed, and missing letters added. The suggested text was added, but it contains some weird references instead of the suggested ones. The reference on Strongyloides was replaced by some irrelevant one; similarly, the reference on Blastocystis was replaced by a paper that only marginally deals with Blastocystis itself. Remove the text likely generated by LLM (the presence of em-dashes, and unnecessary words, such as “Finally”, etc., and unusual words, such as “plausible” suggests use some AI-generated text). Add a real discussion of your results. Include a Statistics section and analyze your data using relevant statistical methods.

R/ We sincerely appreciate the reviewer’s thoughtful comment. In response, we have carefully revised the manuscript by reviewing and correcting all references to ensure that each cited work is relevant, accurate, and properly formatted. In addition, we removed residual Spanish words and typographical inconsistencies throughout the text. We also incorporated the suggested references to strengthen the discussion, emphasizing the relevance of metataxonomic approaches within a One Health framework. We made every effort to ensure that the discussion fully addresses the findings obtained in this study and their broader implications. The revised section now includes the following paragraph:

*Intestinal protozoa and nematode infections occur through interactions between humans, their domestic animals—both companion and livestock— and the surrounding environment. Molecular studies already show parasite exchange across species barriers. Comparative analyses of Strongyloides 18S and cox1 sequences from humans and dogs have revealed two major S. stercoralis lineage, one apparently restricted to canines, and another shared among dogs, cats, humans, and non-human primates (56). These findings indicated a limited yet plausible zoonotic link, though the direction and geographic extent of transmission remain uncertain, underscoring the need for co-sampling of hosts and population genetic analyses to clarify cross-species dynamics. Human-associated Blastocystis subtypes (ST1-ST3) are frequently detected in cohabiting pets and their owners, suggesting either bidirectional transmission or exposure to shared environmental sources (57). Given that most studies are cross-sectional, current evidence provides only limited insights into parasite transmission, highlighting the need for high-resolution genotyping, longitudinal sampling, and environmental assessments. Similarly, genetic analyses show extensive overlap among soil-transmitted helminths in humans and animals, suggesting shared transmission cycles rather than exclusively human reservoirs (8). These findings indicate the need for integrated parasite control and management to mitigate zoonotic and environmental risks. Monitoring and preventive measures across veterinary, medical, and public health sectors are critical to reduce cross-species transmission, but current evidence reveals gaps in treatment, evaluation and coordinated efforts involving health and veterinary professionals, researchers, industry, and governmental authorities (58). In this context, metataxonomic approaches offer a powerful tool to advance One Health investigations, by providing high-resolution data on parasite diversity, geographic distribution, and potential reservoirs, supporting informed, evidence-based interventions across humans, animals, and the environment.*

We also appreciate the reviewer’s observation regarding language and style. The manuscript has been thoroughly reviewed and edited to improve clarity and consistency with the formal scientific tone required by PLOS ONE. Unnecessary words and stylistic elements have been removed to ensure a clear and objective presentation of the findings.

A dedicated section has been added to describe the statistical analyses performed. These analyses correspond to a preliminary exploratory assessment, which we consider appropriate for the current scope of the study and sufficient to support the interpretation of the data. More advanced statistical analyses will be incorporated in future studies as additional datasets become available. Here is the paragraph included in the methodology section:

***Statistical analysis***

*Descriptive analyses were performed to compare the detection outcomes obtained by microscopy and metataxonomic (NGS-based) approaches. The presence or absence of each parasite taxon was summarized as qualitative variables, and detection frequencies were calculated as the proportion of positive samples per method. Simple concordance rates between both diagnostic approaches were also calculated to provide a preliminary measure agreement. Results were visualized using comparative bar plots generated in R (v4.3.1) with the ggplot2 package. No inferential statistical tests were applied, as the objective of this preliminary study was to provide an exploratory, qualitative comparison of diagnostic performance. More comprehensive statistical analyses could be performed in future studies with larger sample sizes.*

Furthermore, we have made a minor modification to the introductory paragraph of the Results section to clearly indicate that this work represents a preliminary comparison between traditional microscopy and metataxonomic approaches for parasite detection. Here is the modified paragraph:

*This study aimed to provide a preliminary comparison of parasite detection in human fecal samples using two approaches: traditional smear microscopy performed by trained parasitologists, and a metataxonomic method targeting the V4 hypervariable region of the eukaryotic 18S rDNA. Previous tests confirmed that the primers used in this study are effective for detecting protists and nematodes but do not amplify DNA from cestodes and trematodes. As a result, these groups were excluded from the scope of our analysis. Accordingly, our study focused on the detection of human intestinal protists and nematodes.*

In addition, the abstract has been revised to reflect the exploratory nature of the study, including the challenges encountered with certain taxa, the differential detection by each method, and the limitations of current primers and DNA extraction techniques. This adjustment ensures that the manuscript accurately conveys the scope and preliminary character of the study. Here is the modified abstract:

*Intestinal parasites continue to pose a significant public health burden in low- and middle-income countries and are increasingly recognized in developed regions. Traditional diagnostic methods, primarily based on microscopy, remain widely used despite limitations in sensitivity and taxonomic resolution. In this exploratory study, we applied a next-generation sequencing (NGS)-based metataxonomic approach, integrated with classical phylogenetic methods, to characterize intestinal parasites in rural Colombian populations. We compare its performance with conventional microscopy, focusing on both protist and geohelminth detection. Metataxonomics outperformed microscopy in detecting Strongyloides stercoralis and enabled precise species and subtype level assignment for Blastocystis and Entamoeba spp., revealing frequent mixed infections. Microscopy detected Trichuris trichiura, Giardia, Cyclospora, and Chilomastix more effectively, highlighting limitations of current primers and DNA extraction methods. Cystoisospora was only identified by NGS. These results demonstrate the utility of NGS-based metataxonomics for broad parasite detection while emphasizing areas for methodological improvement and providing a foundation for future, larger-scale studies.*

We trust that these revisions address the reviewer’s concerns and enhance the overall clarity, rigor, and scientific value of the manuscript.
